# Use of Point-of-care Haemoglobin Tests to Diagnose Childhood Anaemia in Low-and Middle-Income Countries: A Systematic Review

**DOI:** 10.1111/tmi.13957

**Published:** 2023-12-03

**Authors:** Rebecca Brehm, Annabelle South, Elizabeth C George

**Affiliations:** 1 Institute of Clinical Trials and Methodology, UCL, London, UK; 2 Medical Research Council Clinical Trials Unit (MRC CTU) at University College London, London, UK

**Keywords:** Point-of-care testing, haemoglobin, anaemia, systematic review, low-and middle-income countries, children

## Abstract

**Objectives:**

Anaemia is a major cause of mortality and transfusion in children in Low- and Middle-Income Countries (LMICs), however current diagnostics are slow, costly, and frequently unavailable. Point-of-care haemoglobin tests (POC(Hb)Ts) could improve patient outcomes and use of resources by providing rapid and affordable results. We systematically reviewed the literature to investigate what, where and how POC(Hb)Ts are being used by health facilities in LMICs to diagnose childhood anaemia, and to explore challenges to their use.

**Methods:**

We searched a total of nine databases and trial registries up to 10^th^ June 2022 using the concepts: anaemia, POC(Hb)T, LMIC and clinical setting. Adults ≥21 years and literature published >15 years ago were excluded. A single reviewer conducted screening, data extraction and quality assessment (of diagnostic studies) using QUADAS-2. Outcomes including POC(Hb)T used, location, setting, challenges and diagnostic accuracy were synthesised.

**Results:**

Of 626 records screened, 41 studies were included. Evidence is available on the use of 15 POC(Hb)Ts in hospitals (n=28, 68%), health centres (n=9, 22%) and clinics/units (n=10, 24%) across 16 LMICs. HemoCue (HemoCue AB, Ängelholm, Sweden) was the most used test (n=31, 76%). Key challenges reported were overestimation of haemoglobin concentration, clinically unacceptable limits of agreement, errors/difficulty in sampling, environmental factors, cost, inter-observer variability, and supply of consumables. Five POC(Hb)Ts (33%) could not detect haemoglobin levels below 4.5g/dl. Diagnostic accuracy varied, with sensitivity and specificity to detect anaemia ranging from 24.2-92.2% and 70-96.7%, respectively.

**Conclusions:**

POC(Hb)Ts have been successfully utilised in health facilities in LMICs to diagnose childhood anaemia. However, limited evidence is available, and challenges exist that must be addressed before wider implementation. Further research is required to confirm accuracy, clinical benefits, and cost-effectiveness.

## Introduction

Anaemia is a major global health problem, affecting over 1.8 billion people worldwide.^(1, 2)^ The condition is characterised by reduced blood haemoglobin (Hb), resulting in increased morbidity and mortality. Definitions vary by age, with anaemia and severe anaemia classified as Hb<11g/dl and Hb<7g/dl in children aged 6-59 months.^(2)^ Prevalence and years lived with disability are highest in Sub-Saharan Africa and South Asia where nutrient deficiencies, infectious diseases and haemoglobinopathies are common.^(1, 3)^ Children under five years of age are most vulnerable, with an estimated prevalence of 56.5% in Low-and Middle-Income Countries (LMICs).^(4)^

Severe anaemia is life-threatening and accounts for many hospital admissions in Sub-Saharan Africa. A large randomised controlled trial (RCT) investigating fluid bolus on mortality in hospitalised African children with severe infection, found 33% of presented children had Hb level <5g/dl and this resulted in increased mortality (FEAST).^(5, 6)^ Severe anaemia often requires emergency blood transfusion to restore Hb levels. However, this requires efficient diagnosis and availability of donated blood for effective treatment. This poses significant challenges in LMICs, where laboratory analysis is often lengthy, and stock-outs of blood are frequent.^(7)^ Delays in transfusions are common.^(8)^ Results from FEAST show that 52% of severely anaemic children died when not transfused within eight hours, with 90% of deaths occurring within 2.5 hours.^(5, 6)^ Therefore, prompt transfusion is critical to save lives.

Haematology analysers are the routine diagnostic method used to diagnose anaemia. However, equipment is expensive, requires electricity, trained personnel, and regular supply of reagents. This leads to them being often unavailable in LMICs, resulting in inaccurate diagnosis by clinical assessment and inappropriate use of transfusion.^(9–11)^ Point-of-care haemoglobin tests (POC(Hb)T) have been developed to help address these issues. These tests should be Affordable, Sensitive, Specific, User-friendly, Rapid and Robust, Equipment-free and Deliverable, according to World Health Organisation (WHO) ASSURED criteria.^(12)^ They are less invasive and provide immediate results.^(13)^

POC(Hb)Ts have been shown to be valuable in a range of settings.^(14–22)^ The TRACT trial found no significant difference in mortality between immediate and triggered transfusion (by new signs of severity or Hb<4g/dl) after 28 days (Hazard Ratio 0.54; 95% CI: 0.22-1.36; p=0.19), reducing transfusion requirements by 60% and saving valuable resources.^(22)^ These results led to development of a treatment management algorithm to improve clinical practice.^(23)^ However, this approach requires Hb testing every eight hours (first 24 hours), and at 48 hours.

Although several POC(Hb)Ts have been developed and evaluated in recent years, it is unclear to what extent POC(Hb)Ts have been employed to diagnose anaemia in underserved populations. Understanding where and what POC(Hb)Ts are currently used by health facilities and the barriers to their use, will help guide work to improve their availability and allow safe implementation.

The aim of this study was to conduct a systematic review to explore and summarise available evidence on POC(Hb)T use in children in LMICs. Using data from published literature and trial registries, we address the following questions: what, where and how are POC(Hb)Ts being used by health facilities in LMICs to diagnose childhood anaemia, and are there challenges to their use? To the best of our knowledge, our systematic review was the first to address these questions and therefore provides invaluable evidence for policymakers.

## Methods

### Literature search

We conducted a systematic review, reported in line with the Preferred Reporting Items for Systematic Reviews and Meta-Analyses (PRISMA) 2020 guidelines.^(24)^ We aimed to identify all published and unpublished literature using POC(Hb)Ts in children in LMICs. We searched six bibliographic databases: MEDLINE, EMBASE and Global Health via OVID, Web of Science, LILACS, and Cochrane Central Register of Controlled Trials. Clinical trial registries (WHO International Clinical Trials Registry Platform and ClinicalTrials.gov) were searched for unpublished trials. ProQuest Dissertations and Theses were also searched. Entire platforms were searched up to 10^th^ June 2022 and English language filters applied. Relevant journals, articles and authors were manually searched to identify missing literature; this included searching reference lists of included studies.

Search terms were based on four key concepts: anaemia, POC(Hb)T, LMIC and clinical setting. LMIC filters were provided by Cochrane Collaboration and updated according to World Banks Classification 2022.^(25), (26)^ Full details of the search strategy are outlined in Table S1.

### Selection criteria

We included all RCTs and observational studies using POC(Hb)Ts to diagnose anaemia in children (aged 0-20 years) attending health facilities, published/registered within the last 15 years. Reviews, at-home POC Hb testing, testing from non-blood samples, high-income countries, and non-English or non-full text publications were excluded, as were studies not performing POC(Hb)Ts immediately at the site of care (laboratory or delayed sample analysis). There were no restrictions based on child presentation or characteristics to ensure generalisability in the paediatric population.

### Data extraction

Results were exported to Endnote 20 and Rayyan systematic review management software. Duplicates were removed and further checked manually. The primary reviewer (RB) double-screened titles and abstracts for relevance using pre-specified inclusion/exclusion criteria. Potentially eligible studies were further screened by full-text assessment. Any uncertainties on study eligibility were discussed and resolved by consensus with co-authors (ECG, AS).

We extracted: study characteristics, location, setting, POC(Hb)T(s) used, sample, prevalence of mild/moderate/severe/overall anaemia or mean Hb concentration, diagnostic accuracy and challenges to test use reported by study authors. All data were collected using three piloted data extraction tools (study characteristics, challenges, and diagnostic accuracy) created in Microsoft Word (Table S2, S3 and Table 3).

### Quality assessment

The primary reviewer (RB) assessed risk of bias (RoB) and applicability concerns using an adapted QUADAS-2 tool (Table S4).^(27)^ Due to limited evidence on HemoCue (HemoCue AB, Ängelholm, Sweden) device accuracy in this population and setting, studies using HemoCue as reference standard were judged high RoB for domain three. “Unclear” judgement was only made when insufficient evidence was reported.

### Data analysis

Extracted data were synthesised from all included studies and summarised in groups to answer the review question: study characteristics, POC(Hb)Ts, location/setting and challenges. Median and interquartile-range (IQR) were calculated for test sensitivity and specificity using Microsoft Excel. Due to differences in cut-offs used to define anaemia and severe anaemia across included studies and age groups, we summarised data according to how it was reported in papers (severe or overall anaemia) rather than using WHO definitions. For diagnostic studies, test sensitivity and specificity were summarised by including cut-offs of Hb<5g/dl and Hb<7g/dl for diagnosis of severe anaemia. Meta-analysis was not conducted due to insufficient data available for individual POC(Hb)Ts.

## Results

### Study characteristics

**Figure 1:**
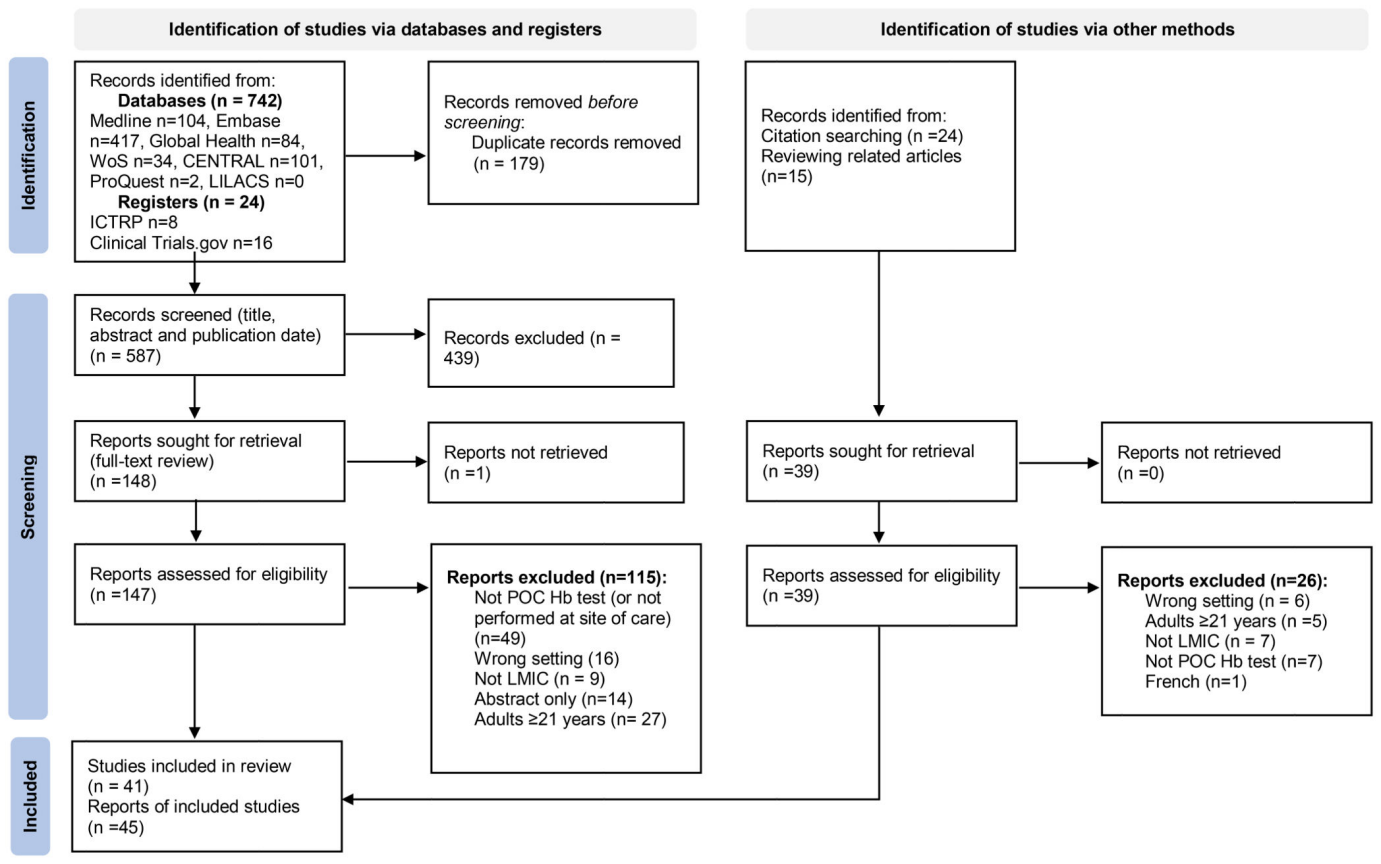
PRISMA 2020 Flow Diagram of Study Selection. Four additional reports were identified for TRACT (n=2) and FEAST (n= 2) trials to give a total 45 reports and 41 included studies. Common reasons for exclusion due to wrong setting included at-home testing. Common reasons for exclusion due to not point-of-care haemoglobin test included use of laboratory analysers or other point-of-care tests for malaria and Sickle Cell Disease. n= number. WoS = Web of Science. CENTRAL = Cochrane Central Register of Controlled Trials. LILACS = Latin American and Caribbean Health Sciences Literature. ICTRP = International Clinical Trials Registry Platform. ProQuest = ProQuest Dissertations and Theses. POC = Point-of-care. Hb = Haemoglobin. LMIC = Low-and Middle-income Country.

742 records were identified from bibliographic databases and 24 from trial registries, (Figure 1). 179 duplicates were removed and 587 records were title and abstract screened. 439 records were excluded, leaving 148 records for full-text assessment. 39 additional records were identified from reviewing related articles and citation lists. In total, 45 records from 41 studies met inclusion criteria and were included in our review.^(3, 5, 6, 13, 15, 19, 22, 28–65)^ Figure 1 shows reasons for exclusions.

Of 41 included studies, 28 (68%) were observational (cross-sectional n=10, case-control n=3, cohort n=1, diagnostic n=14). 12 studies were RCTs (29%) and one study did not report study design.^(48)^ POC(Hb)Ts were implemented in 25 studies (61%) and assessed in 16 (39%). Of studies that assessed POC(Hb)Ts, 14 were diagnostic, one was retrospective, assessing user experiences of POC(Hb)T, and one assessed use as part of an electronic algorithm.^(13, 15, 19, 29, 31, 32, 42, 47, 49, 50, 54, 55, 58, 60, 62, 63)^ Sample size ranged from 67-3,983 children undergoing POC Hb testing. Age ranged from 0-20 years. Only six studies included older children (>15 years) (15%).^(32, 35, 50, 51, 54, 60)^ One study did not report specific age of included children.^(47)^ Four studies also included adults.^(32, 35, 50, 51)^ Overall anaemia prevalence ranged from 11.9-100% (n=16). Prevalence of mild, moderate, and severe anaemia ranged from 2.7-52.2% (n=9), 2.5-60.2% (n=10) and 0.8-52.1% (n=14). Mean Hb concentration measured by POC(Hb)Ts ranged from 3.6-12.8g/dl (n=24). Study characteristics are shown in Table S2.

### POC(Hb)Ts

In total, 15 different POC(Hb)Ts were used (Table 1): 13 (87%) were invasive and required whole blood samples, of which 11 were electronic devices. Two were non-invasive electronic devices (13%). All devices were portable, calibrated and used either changeable or rechargeable batteries, providingresults within two minutes.

HemoCue devices were the most used test (n=31, 76%) and of papers reporting the specific model used was HemoCue301 (n=11, 27%). Six studies used colour-based tests (Haemoglobin Colour Scale (HCS) (COPACK, Germany) n=4, novel-assay n=2).^(29, 47, 55, 60, 63, 65)^ Nine studies used multiple tests.^(15, 29, 32, 37, 49, 55, 58, 63, 65)^ Of studies using invasive tests (n=41), most used capillary sampling (n=19). Eight studies used venous sampling and five studies used both capillary and venous samples. Nine studies did not report the sample type used.^(3, 22, 30, 34, 36, 42, 48, 53, 65)^ All invasive tests, excluding I-STAT (Abbott Park, Illinois) required a sample volume of 15μl or less (Table S5).

### Location and Setting

**Figure 2:**
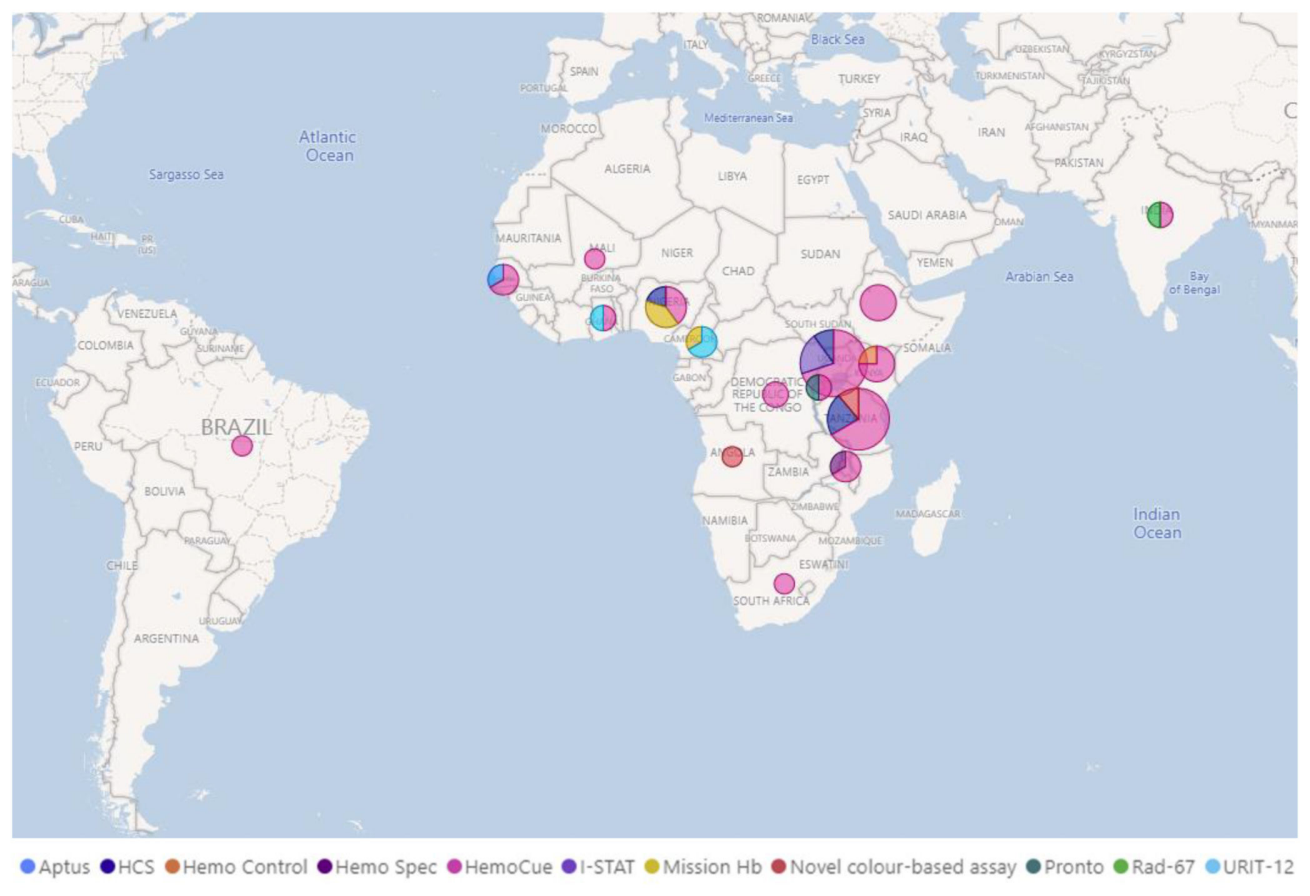
Map Showing where POC Hb Tests have been used by Health Facilities in Children in LMICs. Size of pie chart represents total number of tests and studies. Uganda (HemoCue n=7, I-STAT n=2, HCS n=1), Tanzania (HemoCue n=6, HCS n=2, Novel colour-based assay n=1), Nigeria (HemoCue n=2, Mission Hb n=2, HCS n=1), Ethiopia (HemoCue n=4), Kenya (HemoCue n=3, Hemo Control n=1), Gambia (HemoCue n=2, Aptus n=1), Cameroon (URIT-12 n=2, Mission Hb n=1), Malawi (HemoCue n=2, Hemo Spec n=1), Democratic Republic of Congo (HemoCue n=2), Ghana (HemoCue n=1, URIT-12 n=1), Rwanda (HemoCue n=1, Pronto n=1), India (HemoCue n=1, Rad-67 n=1), Mali (HemoCue n=1), Angola (Novel colour-based assay n=1), South Africa (HemoCue n=1) and Brazil (HemoCue n=1).

Most studies were conducted in Sub-Saharan Africa (n=39, 95%), across 14 African countries, (Figure 2). Two trials included more than one country.^(5, 22)^ One study was conducted in Asia (India) and one in South America (Brazil).^(58, 59)^

28 studies were conducted in hospitals (68%) and Nine in health centres (22%).^(35, 36, 39, 40, 42, 51, 56, 62, 63)^ Eight studies were conducted in health clinics (20%). ^(29, 30, 39, 43, 49, 57, 61, 64)^ Two studies were conducted in basic health facilities (5%).^(59, 65)^ Six studies were multifacility, conducted in hospitals, health centres and/or clinics.^(36, 39, 42, 51, 56, 64)^ One study included a dispensary.^(56)^ Seven studies included rural health facilities.^(3, 30, 43, 48, 49, 62, 63)^

Use of POC(Hb)Ts varied across included studies: to assess diagnostic accuracy (n=14, 34%), assess anaemia prevalence or associated factors/infections (n=13, 32%), assess Hb levels during intervention follow-up (n=6, 15%), assist triage of sick children (n=3, 7%), guide and monitor transfusion in children with severe anaemia (n=3, 7%), assess trial eligibility (n=1, 2%) and assess effect of anaemia on school performance (n=1, 2%).

### Challenges

13 studies (32%) reported challenges to POC(Hb)T use.^(13, 15, 19, 29, 31, 32, 47, 49, 50, 54, 55, 58, 60)^ Overestimation of Hb concentration (n=5) and errors in sampling and environmental factors (n=5) were the most frequently reported challenges. Other challenges included cost of device, consumables and training, difficulty in obtaining measurement, inter-observer variability, supply and stability of consumables, maintenance, and device failure (Table 2).

Diagnostic accuracy data were available for 10 POC(Hb)Ts in 11 of 14 diagnostic studies, (Table 3).^(15, 19, 29, 31, 47, 49, 54, 55, 58, 60, 63)^ Accuracy data were not extracted in three studies due to pooled data with adults and children (n=2) and use of a non Hb-measuring tool (n=1).^(32, 50, 62)^ Five studies reported accuracy results were clinically unacceptable (Table 2).^(15, 29, 31, 32, 49)^ Test performance varied across studies. Sensitivity to detect anaemia and severe anaemia ranged from 24.4-92.2% (median 74%, IQR:32.8, n=4) and 10-92.2% (median 83.7%, IQR:54.4, n=7), respectively. This variability decreased (64-92.2% (n=2) and 84.2-91% (n=3)) when limited to invasive devices.^(15, 19, 54, 58)^ Specificity to detect anaemia and severe anaemia ranged from 70-96.7% (median 84.7%, IQR:6.1 n=4) and 74.5-100% (median 93.2%, IQR:16.5, n=7), respectively.

Mean difference/bias between POC(Hb)Ts and reference ranged from -0.34 to 2.49g/dl (n=8), with lower and upper limits of agreement (LOA) ranging from -0.17 to -3.4g/dl and 1.3 to 5.2g/dl, respectively (n=7). Five studies reported percentage of Hb values within 1g/dl of reference standard.^(15, 29, 47, 49, 63)^

### Quality assessment

We detected high RoB for at least one domain in nine of 11 diagnostic studies (Table 3).^(15, 19, 29, 31, 49, 55, 58, 60, 63)^ No studies showed concerns for applicability. Incomplete reporting resulted in unclear judgement for one or more domains in nine studies.^(15, 19, 31, 47, 49, 54, 55, 58, 60)^ Flow and Timing domain showed the greatest proportion of high RoB due to some enrolled patients missing from final analyses.^(15, 19, 29, 31, 55, 60, 63)^ Five studies were judged high RoB for domain three due to HemoCue as comparator/reference method.^(29, 31, 49, 55, 63)^ Details of the RoB assessment and rationale are presented in Figure S1 and Table S6.

## Discussion

We systematically reviewed the literature to explore POC(Hb)T use in children in LMICs. Using data from published literature and trial registries, we found evidence on the use of 15 different POC(Hb)Ts by health facilities across 16 LMICs in the last 15 years to diagnose childhood anaemia; from 41 studies. 39 studies (95%) were conducted in Africa, indicating little evidence is available outside this region. We found that, to date, HemoCue is the most widely utilised test in this population and setting. Our results represent a relatively small proportion of commercially available and assessed POC(Hb)Ts, suggesting limited evidence is available in children.^(17, 66–70)^ Imperfect diagnostic accuracy, sampling errors, environmental factors and costs were key challenges reported for POC(Hb)T use.

Five POC(Hb)Ts (33%) could not detect Hb levels below 4.5g/dl. This is important because many children with severe anaemia in LMICs have Hb<4g/dl^(3, 71)^ and, for uncomplicated severe anaemia, WHO guidelines recommend blood transfusions only for children with Hb<4g/dl. Three studies in our review reported mean Hb concentration by POC(Hb)T <4g/dl and five additional studies reported Hb values below this level.^(3, 5, 33, 37, 44, 47, 50, 55)^ The WHO guidelines for the management of children with severe anaemia and the transfusion algorithm developed based on results from TRACT use a cut-off of Hb<4g/dl to determine which children require transfusion in the absence of other severity signs.^(23, 72)^ Therefore, Mission Hb (ACON Laboratories, USA), I-STAT, Aptus (Entia, UK), Rad-67 (Masimo, USA) and Pronto (Masimo, USA) are not currently suitable for identifying severe anaemia in this population. It is worth noting however, that I-STAT, Rad-67 and Pronto measure additional parameters besides Hb and haematocrit, increasing their utility. URIT-12 (URIT, China) and HCS have a Hb cut-off of 4g/dl and so accuracy detection at this level must be further investigated.

We identified several challenges to POC(Hb)T use need addressing before wider implementation. No POC(Hb)T showed excellent diagnostic accuracy across all measurements and therefore may not meet all ASSURED criteria.^(12)^ Five studies reported overestimation of Hb concentration in eight POC(Hb)Ts (Table 2).^(15, 29, 32, 49, 58)^ This is vital since it could result in misclassification of severity of anaemia and therefore prevent truly eligible children from receiving a lifesaving transfusion or appropriate treatment. In contrast, underestimation of Hb levels by POC(Hb)Ts has been reported in children, causing unnecessary use of scarce resources and exposure of children to transfusion-related risks.^(73, 74)^ A previous review of HemoCue found underestimation of Hb most frequently reported, with few studies reporting overestimation of Hb concentration.^(75)^ These conflicting findings could be explained by variations in child Hb level.^(15, 76–78)^

Similarly, wide LOA means estimated Hb values could span all categories of anaemia. Our results show seven diagnostic studies and eight POC(Hb)Ts exceeded the clinically acceptable accuracy of upper and lower LOA within 1g/dl (Table 3).^(15, 19, 29, 47, 49, 54, 58)^ This suggests within-subject variation is too large to provide a clinically meaningful diagnosis. These results are in line with a previous systematic review including adults and children in mixed settings.^(79)^ However, a different review has shown clinically acceptable LOA.^(80)^ Clinicians should be aware of these LOA when basing clinical decisions solely on these Hb estimations. We found substantial variation in test sensitivity to detect anaemia and severe anaemia, with lowest values reported for HCS and Rad-67.^(29, 58)^ This is contrary to a previous review that identified lowest sensitivity to detect anaemia for HemoCue301 (22.6%) and highest sensitivity for HCS (99.3%) out of 6 POC(Hb)Ts.^(81)^ Our median values for test sensitivity and specificity to detect anaemia were moderate, suggesting some children would receive a false negative result.

Variation in test performance across studies in our review and with previous literature has several possible explanations. Firstly, nine studies in our review were judged high RoB for at least one domain.^(49, 58) (15, 19, 29, 31, 55, 60, 63)^ Differences in anaemia prevalence, geographical factors (temperature, altitude, and humidity), reference test, transportation and storage of consumables, sampling technique and training could also explain discrepancy.^(15, 82, 83)^ Three studies did not use thresholds for severe anaemia defined by WHO for the assessment of sensitivity and specificity (Hb<5g/dl), limiting evidence synthesis and contributing to disparity.^(19, 29, 55)^ Lastly, two included studies used different sources of sample for the reference method and POC(Hb)T, and five included diagnostic studies used venous samples for POC(Hb)T.^(15, 19, 47, 49, 54, 58, 60)^ This could explain variation due to known differences in capillary and venous blood.^(84)^

These real-life factors pose a challenge to POC(Hb)T use. There is a need for standardised training protocols to reduce errors in sampling technique and interpretation of colour-based tests. Competency of staff and therefore performance should increase as these tests become routine practice. Our findings also suggest five POC(Hb)Ts used may not be suitable for use in some LMICs due to possible device failure at high temperature (>30°C) (Table 1).^(15, 19, 32, 35, 39, 42, 50–52, 57)^

Moreover, we identified analyser costs and lack of supply of consumables as challenges to POC(Hb)T use. Although upfront costs are relatively high, particularly for non-invasive and HemoCue devices, it is the recurrent, per test costs that pose an obstacle to sustained use in LMICs and could explain the lack of supply of microcuvettes and cartridges.^(85)^ Total cost of POC tests must be weighed against their benefits such as earlier diagnosis, reduced morbidity and mortality, improved patient satisfaction and decrease of unnecessary referrals, and additional testing.^(86)^ Use of POC(Hb)Ts with electronic decision support algorithms could enhance their cost-effectiveness in triage of sick children. However, only one included study adopted this approach and therefore this requires further research.^(42)^ The novel colour-based assay offers a significant cost advantage at 0.26USD per test, however further evidence on its use is required. Other affordable technologies, such as smartphone-based colorimetry are at early stages of development for identifying severe anaemia in LMICs.^(87)^

Key strengths of our review were wide inclusion criteria and adherence to systematic review methods. This allowed a comprehensive synthesis of POC(Hb)T use in this population and setting. Ongoing trials were included and therefore reduced publication bias. Furthermore, we assessed RoB and applicability of included diagnostic studies to evaluate the reliability and validity of findings on POC(Hb)T performance.

Our review has limitations. A single reviewer (RB) screened results, adapted QUADAS-2 tool and judged study inclusion eligibility and RoB and applicability concerns. However, records were screened twice to minimise risk of missing relevant articles and uncertainties were resolved by consensus with co-authors (ECG, AS). We searched a restricted number of databases and trial registries; however they were the largest and most renowned databases, specific to the subject area. Other methodological limitations include exclusion of reviews, non-full-text or non-English articles and studies published over 15 years ago. Only 13 studies (32%) reported challenges to POC(Hb)T use and therefore our review may be subject to reporting bias if not all challenges were reported. We only assessed data from published literature and trial registries. Clinical trials/studies may not represent the total use of POC(Hb)Ts and may be atypical due to trial funding and supply of tests and consumables.

Another limitation of our work is our focus solely on tests used in children. Our decision to focus on tests available for children was driven by the requirement to be able to detect lower haemoglobin levels in children than is needed for adults, given the different thresholds for defining severe anaemia in these groups. ^(88, 89)^ However, by excluding studies that looked at POC(Hb)Ts used only in adults, we may have missed some relevant studies that may be relevant for children as well. Different studies used different definitions of anaemia and severe anaemia, and we report the definitions used, as we were unable to convert this to standardised definitions. There is debate at the moment about what thresholds should be used ^(88, 89)^, and WHO guidelines for malaria differ in the thresholds used to those in guidelines for care of children in hospital^(72, 90)^.

### Further research

Diagnostic accuracy data for this population and setting was available for only 10 POC(Hb)Ts. This finding is critical, since POC(Hb)Ts must be validated in the population and setting of their intended use before wider adoption. Further high-quality research on test accuracy, particularly using capillary samples, is warranted to assess performance in various settings and address discrepancy between studies. There is currently insufficient data to conduct meta-analysis on individual POC(Hb)Ts in this population and setting. Further research should ensure data and results can be combined with previous studies for meta-analysis.

Very few studies used POC(Hb)Ts in guiding and monitoring transfusion.^(5, 22, 37)^ Therefore, further research is essential to evaluate use with the transfusion algorithm and impact on patient-centred outcomes, time to transfusion and usage of blood supply. Further research is also required to understand the clinical and resource implications of under/overestimating Hb levels, especially near the cut-off for severe anaemia. The challenges identified in our review also stress the need for further development of some POC(Hb)Ts and standardised training procedures. For example, by expanding detection ranges to include lower Hb levels and to enable higher operating temperatures.

## Conclusions

In conclusion, 15 POC(Hb)Ts have been successfully utilised in health facilities across 16 LMICs to diagnose childhood anaemia of various aetiologies. However, several challenges to their use exist and must be addressed. We found HemoCue301, HemoCue801 and HemoControl (EKF Diagnostics, UK) offer the most suitable Hb detection ranges and operating temperatures (<40°C) for use in this setting. However, we identified no evidence on diagnostic accuracy for HemoCue801 and HemoControl. We therefore recommend HemoCue301 as the best available POC(Hb)T to diagnose childhood anaemia in LMICs, based on available evidence. However, imperfect diagnostic accuracy is a drawback and must be weighed against benefits in costs, safety, convenience, and improved clinical outcome. Further research is essential to confirm these benefits and diagnostic accuracy in these settings. Routine use of POC(Hb)Ts may significantly reduce child mortality in LMICs, where laboratory analysers are often unavailable and anaemia prevalence is high.

## Supplementary Material

Supplementary material

## Figures and Tables

**Table 1:** Description and Summary of POC Hb Tests Used.

POC Hb test	Principle method	Consumables	Hb detection range (g/dL)	Operating conditions (temperature; humidity)	Total studies (n)	Reported Study Setting		
						Hospital (n)	Health Centre (n)	Clinic/basic facilities (n)
**Total studies (n)**					41*	28*	9*	10*
**Invasive**								
**HemoCue – Exact model not reported** (HemoCue AB, Ängelholm, Sweden)					12 (29%) ^(3, 29, 30, 33, 34, 37, 40, 41, 43, 48, 59, 64)^	7 ^(3, 33, 34, 37, 41, 48, 64)^	1 ^(40)^	5 ^(29, 30, 43, 59, 64)^
**HemoCue 301** (HemoCue AB, Ängelholm, Sweden)	Absorbance at Hb/HbO2 isosbestic point; transmittance measured at 506nm and 880nm	Reagent-free microcuvettes	0-25.6	10-40°C; ≤90%	11 (27%) ^(5, 22, 28, 36, 38, 49, 55, 58, 61–63)^	7 ^(5, 22, 28, 36, 38, 55, 58)^	3 ^(36, 62, 63)^	2 ^(49, 61)^
**HemoCue 201+** (HemoCue AB, Ängelholm, Sweden)	Modified azidemethaemoglobin reaction; absorbance measured at 570nm and 880nm	Reagent-containing microcuvettes	0-25.6	15-30°C; ≤90%	4 (10%) ^(15, 39, 42, 52)^	3 ^(15, 42, 52)^	2 ^(39, 42)^	1 ^(39)^
**HCS** (COPACK, Oststeinbek, Germany)	Absorbance of blood on chromatography paper and colour compared to a scale of six shades of red representing Hb levels at 2g/dl intervals	Filter paper test strips	4-14 (Scale at 4,6,8,10,12, and 14)	Not known	4 (10%) ^(29, 55, 63, 65)^	1 ^(55)^	1 ^(63)^	2 ^(29, 65)^
**Mission Hb** (ACON Laboratories, Inc., San Diego, USA)	Modified azidemethaemoglobin reaction measured by reflectance photometry	Reagent-containing test strips, micropipette	4.5-25.6	10-40°C; ≤90%	3 (7%) ^(32, 53, 54)^	3	0	0
**URIT-12** (URIT Medical Electronics, Guangxi, China)	Modified azidemethaemoglobin reaction measured by reflectance photometry	Reagent-containing test strips	4-24	15-30°C; ≤80%	3 (7%) ^(32, 51, 57)^	2 ^(32, 51)^	1 ^(51)^	1 ^(57)^
**I-STAT** (Abbott, Abbott Park, IL)	Measures conductivity and corrects for electrolyte concentration to estimate HCT. Hb then calculated using the formula: Hb (g/dl) = HCT (% packed cell volume) x0.34	Cartridges (EC8+ and CHEM8+)	5.1-25.5	16-30°C; ≤90%	2 (5%) ^(19, 37)^	2	0	0
**HemoCue 201** (HemoCue AB, Ängelholm, Sweden)	Modified azidemethaemoglobin reaction; absorbance measured at 570nm and 880nm	Reagent-containing microcuvettes	0-25.6	18-30°C; ≤90%	2 (5%) ^(35, 65)^	0	1 ^(35)^	1 ^(65)^
**Novel colour-based assay**	Reaction between Hb, hydrogen peroxide and 3,3’,5,5’-TMB that produces a colour change according to Hb concentration: blue (Hb<3g/dl), blue/green (Hb 3-5g/dl), yellow/orange (Hb 5-7g/dl), orange/red (Hb 7-9g/dl) to red (Hb >9g/dl)^(47, 60)^	Reagent tubes and 10μl capillary tube	2.5-9.1	Not known	2 (5%) ^(47, 60)^	2	0	0
**HemoCue 801** (HemoCue AB, Ängelholm, Sweden)	Absorbance of whole blood at Hb/HbO2 isosbestic point; transmittance measured at 506nm and 880nm	Reagent-free microcuvettes	1-25.6	10-40°C; ≤90% (25°C), ≤75% (40°C)	1 (2%) ^(13)^	1	0	0
**HemoCue B-haemoglobin** (HemoCue AB, Ängelholm, Sweden)	Modified azidemethaemoglobin reaction; absorbance measured at 570nm and 880nm	Reagent-containing microcuvettes	0-25.6	15-30°C	1 (2%) ^(50)^	1	0	0
**Aptus** (Entia, London, UK)	Centrifugation and photometry at 515nm, 660nm and 940nm to estimate HCT level. Hb calculated using MCHC x HCT	Microcuvettes	5-25	5-45°C; ≤90%	1 (2%) ^(49)^	0	0	1
**HemoSpec**	Modified azidemethaemoglobin reaction; absorbance measured at 532nm and 650nm	Reagent-containing chromatography paper	Not known	Not known	1 (2%) ^(31)^	1	0	0
**HemoControl** (EKF Diagnostics, Cardiff, UK)	Modified azidemethaemoglobin reaction; absorbance measured at 570nm and 880nm	Reagent-containing microcuvettes	0-25.6	15-40°C; <90%	1 (2%) ^(56)^	1	1	1
**Non-invasive**					**2**	**2**	**0**	**0**
**Rad-67™ Pulse CO-Oximeter® and rainbow® DCI®-mini-Sensor** (Masimo Corporation, Irvine, USA)	Visible and infrared lights (500-1400nm) signalled through capillary bed and sensor detects changes in light absorption. Hb then calculated using a multi-wavelength calibration equation	None	8-17	0-35°C; 10-95%	1 (2%) ^(58)^	1	0	0
**Pronto® device with DCI-mini™ sensors** (Masimo Corporation, Irvine, USA)	Visible and infrared lights (500-1300nm) signalled through capillary bed and sensor detects changes in light absorption. Hb then calculated using a multi-wavelength calibration equation	None	8-17	5-40°C; 5-95%	1 (2%) ^(15)^	1	0	0

Tests presented in order of most used (number of studies). * Six studies were conducted across several settings and nine studies used more than one test and so total number in columns does not equal total number of studies or 100%. HCT = Haematocrit. MCHC = Mean Corpuscular Haemoglobin Concentration. n= Number of studies. Hb = Haemoglobin. HbO2 = Oxyhaemoglobin. IL = Illinois. UK = United Kingdom. USA = United States of America. nm = Nanometre. μl = Microlitre. g/dl = Grams per decilitre. °C = Degrees Celsius.

**Table 2:** Summary of key Challenges Reported.

Challenges	POC Hb test(s)	Number of studies (n=)
Overestimation of Hb concentration	Aptus^(49)^ HemoCue 301^(49, 58)^ HemoCue 201+*^(15)^ _Pronto_^*(15)^ _URIT-12_^**(32)^, Mission Hb^**(32)^, _HCS_^(29)^, Rad-67^(58)^	5
Differences in sampling technique (e.g. milking of finger, pressure, air bubbles, excess blood on back of microcuvettes, insufficient sample volume) or environmental factors can affect results	HemoCue 801^(13)^ HemoCue B-haemoglobin^(50)^ HemoCue 201+^(15)^ HemoCue 301^(58)^ I-STAT^(19)^	5
Clinically unacceptable accuracy	Aptus^(49)^, HemoCue 301^(49)^ HCS^(29)^ HemoSpec^(31)^ Pronto^(15)^ Mission Hb**^(32)^	5
Cost of equipment/disposable consumables, maintenance, or training	HemoCue 201+^(15)^ HemoCue 801^(13)^, HemoCue 301^(55)^ Rad-67^(13, 58)^ Pronto^(13)^	4
Difficulty in obtaining measurement/capillary sample in agitated children– can take longer to take measurement	Rad-67^(58)^, HemoCue 801^(13)^ Aptus^(49)^	3
Underfilling or overfilling of cuvettes/ difficulty handling	Aptus^(49)^, HemoCue 801^(13)^	2
Variability and subjectivity of result interpretation. Difficult to estimate Hb value with high precision to 0.1 or 0.2g/dl	Novel colour-based assay^(47)^ HCS^(29)^	2
Lack of supply of consumables (disposable microcuvettes and cartridges)	I-STAT^(19)^ HemoCue 201+^(15)^	2
Maintenance or regular QC checks to operate correctly	Mission Hb^(54)^ HemoCue 801^(13)^	2
Device failure at high temperature	I-STAT^(19)^	1
Reagent instability	Novel colour-based assay^(60)^	1
Insufficient capillary blood due to multiple POC tests using same finger prick (e.g. malaria rapid diagnostic testing)	HemoCue 801^(13)^	1

Challenges reported for included studies in order of most frequently reported (by study authors). POC = Point-of-care. Hb = Haemoglobin. QC= Quality Control. g/dl = grams per decilitre. * HemoCue 201+ (wicking) and Pronto overestimated Hb concentration for anaemic children only. ** Authors reported overestimation of Hb/clinically unacceptable accuracy based on pooled children and adult data.

**Table 3:** Diagnostic Accuracy of Included POC Hb Tests.

POC Hb test (sample)/Study	Reference/comparator test (sample)	Risk of Bias (A) and Applicability (B)	Sensitivity (95% CI)	Specificity (95% CI)	Correlation coefficients	Bias (95% CI) with 95% LOA (g/dL)	Percentage of samples within Xg/dL
**HemoCue 301**							
Nass *et al*., 2020 ^(49)^ (venous)	Medonic M-Series M16S/M20S haematology analyser (venous)	1. A: 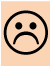 B: 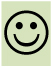 2. A 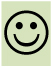 B: 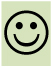 3. A 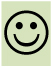 B: 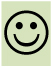 4. A: 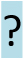	Not reported	Not reported	r_c_= 0.64 (0.57-0.69)	0.81 (0.73-0.88) 95% LOA: -0.17, 1.78	69.4% within 1g/dl
Ramaswamy *et al*., 2021^(58)^ (capillary)	Sysmex XS 1000i haematology analyser (venous)	1. A: 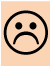 B: 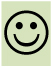 2. A 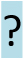 B: 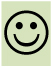 3. A 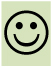 B: 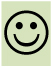 4. A: 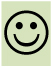	Anaemia (undefined): 92.2% SA (Hb<7g/dl): 90%	Anaemia (undefined): 83.3% SA (Hb<7g/dl): 90.8%	ρ_c_ = 0.90 (p<0.001)	0.27±1.9 95% LOA: -1.4, 1.9	Not reported
**HemoCue 201+**							
Parker *et al*., 2018^(15)^ (venous)	Sysmex KN21 haematology analyser (venous)	1. A: 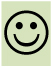 B: 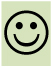 2. A 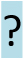 B: 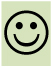 3. A 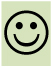 B: 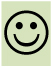 4. A: 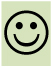	Anaemia (Hb<11g/dl): Wicking: Age 6-59 months: 88%, Age 18-59 months: 89% Gravity: Age 6-59 months: 76%, Age 18-59 months: 64%	Anaemia (Hb<11g/dl): Wicking: Age 6-59 months: 81%, Age 18-59 months: 86% Gravity: Age 6-59 months: 83%, Age 18-59 months: 88%	Wicking: r=0.80 (p<0.001) Gravity: r=0.90 (p<0.001)	Wicking: -0.3 (-0.6, -0.1) 95% LOA: -2.3, 1.7 Gravity: 0.0 (-0.1, 0.2) 95% LOA: -1.4, 1.4	Wicking: 77% within 1g/dl Gravity: 91% within 1g/dl
**HCS**							
Aldridge *et al*., _2012_^(29)^ (capillary)	HemoCue (capillary)	1. A: 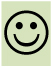 B: 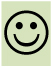 2. A 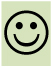 B: 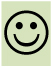 3. A 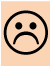 B: 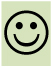 4. A: 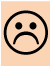	Anaemia (Hb<11g/dl): 33% (29-36) SA (Hb<5g/dl): 14% (0-58)	Anaemia (Hb<11g/dl): 87% (83-91) SA (Hb<5g/dl): 100% (99-100)	Not reported	1.3 (1.2-1.5) 95% LOA: -2.4, 5.1	32% within 1g/dl 60% within 2g/dl
Olupot-Olupot *et al*, 2019^(55)^ (not reported)	HemoCue 301 (capillary)	1. A: 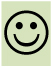 B: 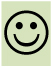 2. A 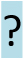 B: 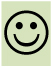 3. A 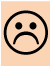 B: 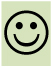 4. A: 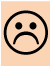	Mild (Hb 10.0-11.9g/dl): 37.5% (8.5-75.5) Moderate (Hb 5.0-9.9g/dl): 51.1% (42.2-60.1) Severe (Hb <5.0 g/dl): 43.1% (34.2-52.3)	Mild (Hb 10.0-11.9g/dl): 82.9% (77.7-87.4) Moderate (Hb 5.0-9.9g/dl): 50.3% (41.5-59.2) Severe (Hb <5.0 g/dl): 95.6% (90.7-98.4)	Not reported	Not reported	Not reported
Ughasoro *et al*., _2019_^(63)^ (capillary)	HemoCue 301 (capillary)	1. A: 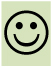 B: 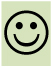 2. A 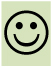 B: 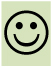 3. A 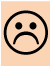 B: 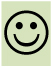 4. A: 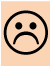	Anaemia (Hb<11g/dl): 89.1%	Anaemia (Hb<11g/dl): 90.2%	Not reported	Not reported	74.7% within 1g/dl
**Mission Hb**							
Olatunya *et al*., _2016_^(54)^ (venous)	Sysmex KX21N haematology analyser (venous)	1. A: 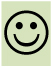 B: 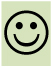 2. A 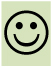 B: 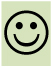 3. A 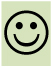 B: 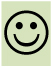 4. A: 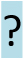	SA (Hb <7g/dl): 84.2% (60.4-96.6)	SA (Hb <7g/dl): 98.6% (92.6-100.0)	ICC: 0.85 (0.75-0.95) CCC: 0.94 (0.92-0.95)	-0.34 (0.99-0.31) (p=0.300) 95% LOA: -2.30, 1.62	Not reported
**I-STAT**							
Hawkes *et al*., _2014_^(19)^ (venous)	Beckman Coulter AcT 5 Diff haematology analyser (CBC) (venous)	1. A: 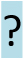 B: 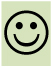 2. A 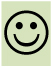 B: 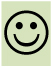 3. A 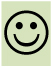 B: 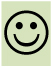 4. A: 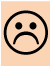	SA (Hb<5g/dl): 91% (77-97)	SA (Hb<5g/dl): 89% (79-92)	r=0.73	-0.18 (3.1-3.5) LOA: -3.4, 3.4	Not reported
**Novel POC colour-based assay**							
McGann *et al*., _2015_^(47)^ (Capillary and venous)	Sysmex KX-21N haematology analyser (venous)	1. A: 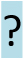 B: 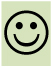 2. A 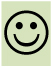 B: 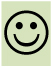 3. A 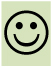 B: 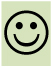 4. A: 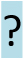	SA (Hb<7.1g/dl): 92.2% (80.3-97.5) Very SA (Hb<5g/dl): 88.9% (50.7-99.4)	SA (Hb<7.1g/dl): 82.9% (65.8-92.8) Very SA (Hb<5g/dl): 98.7% (92-99.9)	r=0.88 (p<0.0001)	0.04±0.6 95% LOA: -1.4,1.3	71.05% within 0.5g/dl 90.7% within 1g/dl
Smart *et al*., _2017_^(60)^ (capillary)	Mindray BC-3200 haematology analyser (venous)	1. A: 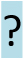 B: 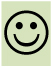 2. A 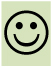 B: 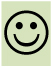 3. A 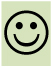 B: 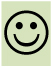 4. A: 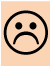	SA (Hb≤7g/dl): 83.2% (78.8-87.0) Very SA (Hb≤5 g/dl): 50.3% (42.0-59.6)	SA (Hb≤7g/dl): 74.5% (69.9-78.6) Very SA (Hb≤5 g/dl): 81.3% (75.5-86.2)	r=0.65	Observer 1: 1.0±0.9 Observer 2: 1.1±0.9	Results within 1g/dl – percentage not reported
**Aptus**							
Nass *et al*., 2020^(49)^ (venous)	Medonic M-Series M16S/M20S haematology analyser (venous)	1. A: 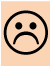 B: 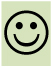 2. A 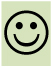 B: 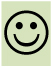 3. A 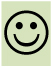 B: 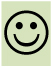 4. A: 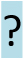	Not reported	Not reported	r_c_= 0.55 (0.46-0.63)	0.69 (0.57-0.81) 95% LOA: -0.92, 2.31	63.3% within 1g/dl
Nass *et al*., 2020^(49)^ (venous)	HemoCue 301 (venous)	1. A: 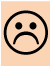 B: 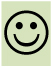 2. A 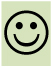 B: 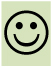 3. A 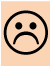 B: 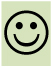 4. A: 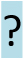	Not reported	Not reported	r_c_=0.69 (0.60-0.76) (venous)	-0.11 (-0.23-0.00) 95% LOA: -1.63, 1.40	83.9% within 1g/dl
Nass *et al*., _2020_^(49)^ (capillary)	HemoCue 301 (capillary)	1. A: 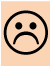 B: 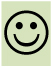 2. A 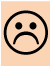 B: 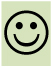 3. A 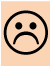 B: 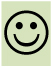 4. A: 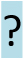	Not reported	Not reported	Not reported	0.32 (0.22-0.42) 95% LOA: -1.90, 2.54	Not reported
**HemoSpec**							
Bond *et al*., 2014^(31)^ (capillary)	HemoCue 201+ (capillary)	1. A: 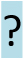 B: 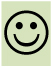 2. A 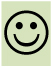 B: 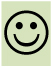 3. A 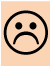 B: 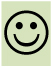 4. A: 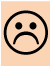	Not reported	Not reported	Not reported	Not reported	86% within 2g/dl
**Rad-67**							
Ramaswamy *et al*., 2021^(58)^	Sysmex XS 1000i haematology analyser (venous)	1. A: 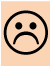 B: 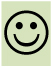 2. A 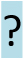 B: 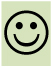 3. A 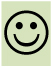 B: 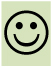 4. A: 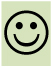	Anaemia (undefined): 24.4% SA (Hb<7g/dl): 10%	Anaemia (undefined): 96.7% SA (Hb<7g/dl): 100%	ρ_c_ =0.30 (p<0.001)	2.49±0.1 95% LOA: -0.3, 5.2	Not reported
**Pronto**							
Parker *et al*., 2018^(15)^	Sysmex KN21 haematology analyser (venous)	1. A: 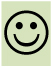 B: 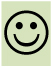 2. A 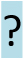 B: 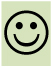 3. A 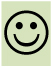 B: 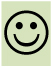 4. A: 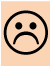	Anaemia (<11g/dl): Age 6-59 months: 66%, Age 18-59 months: 72%	Anaemia (<11g/dl): Age 6-59 months: 70%, Age 18-59 months: 84%	r=0.43 (<0.0001)	-0.2 (-0.5, 0.0) 95% LOA: -2.4, 2.0	65% within 1g/dl 91% within 2g/dl

Test performance data from all included diagnostic accuracy studies. Tests presented in order of most used (same as Table 1). Risk of Bias and Applicability 1. = Domain 1: Patient Selection. 2. = Domain 2: Index Test. 3. = Domain 3: Reference Standard. 4. = Domain 4: Flow and Timing. A= Risk of Bias. B= Applicability Concern. 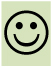 = Low. 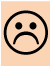 = High. 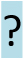 = Unknown. Wicking: Standardised method used to draw capillary blood directly from skin into the microcuvette. Gravity: Non-standardised method where capillary blood is first transferred onto a surface before drawn into the microcuvette. SA = Severe anaemia. POC Hb test = Point-of-care Haemoglobin Test. HCS = Haemoglobin Colour Scale. CI = Confidence Interval. LOA= Limits of Agreement. g/dL = grams per decilitre. r= Pearson’s Correlation Coefficient. ρ_c_ = Lin’s Concordance Correlation Coefficient. ICC = Intraclass Correlation Coefficient. CCC = Concordance Correlation Coefficient. Correlation data rounded to 2 decimal places.
